# Eculizumab Pharmacokinetics and Pharmacodynamics in Patients With Neuromyelitis Optica Spectrum Disorder

**DOI:** 10.3389/fneur.2021.696387

**Published:** 2021-11-03

**Authors:** Pratap Singh, Xiang Gao, Huub Jan Kleijn, Francesco Bellanti, Ryan Pelto

**Affiliations:** ^1^Department of Clinical Pharmacology, Alexion Pharmaceuticals Inc., Boston, MA, United States; ^2^Department of Pharmacometrics, PK/PD M&S, Clinical Development and Translational Sciences, Alexion Pharmaceuticals Inc., Boston, MA, United States; ^3^Certara Strategic Consulting, Oss, Netherlands

**Keywords:** neuromyelitis optica spectrum disorder, pharmacokinetics, pharmacodynamics, complement, eculizumab, autoimmune, exposure-response analysis

## Abstract

**Objective:** To investigate the pharmacokinetics and pharmacodynamics of the approved 900/1,200 mg dosing regimen for the terminal complement component 5 (C5) inhibitor eculizumab in patients with neuromyelitis optica spectrum disorder (NMOSD).

**Methods:** Data were analyzed from 95 patients with aquaporin-4-IgG-positive NMOSD who received eculizumab during the PREVENT study (ClinicalTrials.gov: NCT01892345). Relationships were explored between eculizumab exposure and free complement C5 concentrations, terminal complement activity, and clinical outcomes.

**Results:** Pharmacokinetic data were well-described by a two-compartment model with first-order elimination, and time-variant body-weight and plasmapheresis/plasma exchange effects. Steady-state serum eculizumab concentrations were achieved by Week 4 and were sustained, with serum trough eculizumab concentrations maintained above the 116 μg/ml threshold for complete complement inhibition throughout 168 weeks of treatment in all post-baseline samples from 89% of patients. Complete inhibition of terminal complement was achieved at Day 1 peak and pre-dosing trough eculizumab concentration in nearly all post-baseline samples assessed (free C5 <0.5 μg/ml in all post-baseline samples from 96% of patients; *in vitro* hemolysis <20% in all post-baseline samples from 93% of patients). Kaplan–Meier survival analysis of time to first relapse showed separation of eculizumab-treated patients from those receiving placebo, but no separation based on eculizumab exposure quartile, indicating an optimized dose regimen with maximized efficacy.

**Conclusions:** The approved eculizumab dosing regimen (900/1,200 mg) for adults with aquaporin-4-IgG-positive NMOSD is confirmed by rigorous quantitative model-based analysis of exposure–response. The data demonstrate that eculizumab's mechanism of action translates into clinical effect by achieving rapid, complete, and sustained terminal complement inhibition.

## Introduction

Neuromyelitis optica spectrum disorder (NMOSD) is a rare autoimmune disorder of the central nervous system (CNS), predominantly affecting the optic nerves and spinal cord, and characterized by severe and unpredictable attacks (relapses). These relapses can cause disabling neurologic damage, including weakness, numbness, bowel/bladder dysfunction, pain, paralysis, and blindness (1, 2). The damage is irreversible and its accumulation increases overall mortality (3–6).

The majority of patients with NMOSD (62–88%) test positive for autoantibody immunoglobulin (Ig)G to the water-channel protein aquaporin-4 (AQP4), which is expressed predominantly on the endfeet processes of astrocytes in the CNS (3, 7–9). The binding of anti-AQP4-IgG is one of the key underlying causes of neurologic damage in NMOSD, as the autoantibodies are predominantly of IgG1 subclass and induce activation of the complement cascade. It is thought that anti-AQP4-IgG is generated in the periphery and crosses the blood–brain barrier by passive diffusion from serum (9, 10). The first indication of the role of complement in the pathophysiology of NMOSD came with the demonstration of deposits of perivascular complement components and evidence for the formation of the membrane attack complex in demyelinating lesions in patients with NMOSD (11). Subsequent *in vitro*, animal-model, and clinical studies have provided evidence for activation of the complement pathway in response to the binding of anti-AQP4-IgG to astrocytic AQP4 in the CNS (12–15). The activation results in cleavage of complement protein C5 into C5a (which promotes inflammation) and C5b (which coordinates formation of the membrane attack complex); increased concentrations of terminal complement components (C5a and sC5b-9) have been demonstrated in the cerebrospinal fluid (CSF) of patients with active NMOSD (12, 15). The complement activity rapidly causes damage to astrocytes, as well as permeabilization of the blood–brain barrier, ultimately leading to astrocyte necrosis, demyelination, and neuronal death (11, 16–19).

As complement activation is one of the major determinants of disease pathogenesis in patients with NMOSD, inhibition of terminal complement activation is an effective therapeutic approach (14, 17, 20, 21). This was confirmed in the Phase 3, randomized, double-blind, placebo-controlled, time-to-event PREVENT study in 143 adults with AQP4-IgG-positive NMOSD, in which treatment with eculizumab—a terminal complement inhibitor—significantly reduced the risk of relapse by 94% vs. placebo (*p* < 0.001). Based on results from this study, eculizumab was approved in 2019 in the US, Europe, Japan, and Canada, and in 2020 in Australia, for the treatment of NMOSD in adult patients who are anti-AQP4 antibody positive (22–27).

Eculizumab is a novel, humanized IgG2/4 monoclonal antibody that inhibits terminal complement activation by selectively binding with high affinity to complement component C5, inhibiting its cleavage into C5a and C5b and the subsequent neurologic damage (8, 28). It does not interact with earlier (proximal) components in the complement-activation pathway thus preserving complement functioning at that level, which is required for opsonization and immune complex clearance (29, 30). Eculizumab was derived from a murine anti-human C5 monoclonal antibody and engineered to reduce immunogenicity and the potential to elicit proinflammatory and antigenic responses by substituting with human IgG2 and IgG4 components, which, compared with IgG1 and IgG3, have low potential for activating the complement cascade (29).

Using data from the PREVENT study, the aim of the current analysis was to further characterize the pharmacokinetic and pharmacodynamic properties of eculizumab in patients with AQP4-IgG-positive NMOSD to confirm the rationale, efficacy, and safety of the approved eculizumab dosing regimen in this indication. A model-based approach was used to characterize the population-pharmacokinetic parameters of eculizumab and between-subject variability, to assess potential factors impacting eculizumab pharmacokinetics, and to explore pharmacokinetic/pharmacodynamic relationships for free serum and CSF concentrations and terminal complement activity. Exposure–response relationships were also assessed, as well as the potential immunogenicity of eculizumab.

## Methods

### Study Population and Study Design

This analysis was based on pharmacokinetic, pharmacodynamic, efficacy, and safety data from the randomized, double-blind, placebo-controlled, multicenter, Phase 3 PREVENT study (ClinicalTrials.gov: NCT01892345) of eculizumab in patients with AQP4-IgG-positive NMOSD. The primary endpoint was time to first adjudicated on-trial relapse. Clinical efficacy and safety results have been reported (31).

The PREVENT study population comprised 143 adult patients with AQP4-IgG-positive NMOSD, of whom 96 patients received the now approved dosing regimen of intravenous eculizumab 900 mg weekly (every 7 ± 2 days) for the first four doses (starting on Day 1), followed by 1,200 mg 1 week later at Week 4 (the fifth dose) and then every 2 weeks (14 ± 2 days) thereafter.

The study was conducted in accordance with the Declaration of Helsinki (32), the International Conference on Harmonisation Guidelines for Good Clinical Practice (33), and applicable regulatory requirements. It was approved by the institutional review board at each participating institution. All the patients provided written informed consent before participation.

### Sample Collection

Each intravenous eculizumab infusion was administered over ~ 35 min. Blood samples for measurement of baseline and trough eculizumab concentrations, anti-AQP4 antibody titer, serum free C5 concentrations, and hemolytic activity were taken 5–90 min before infusion of eculizumab (trough concentration; C_trough_) at baseline, Weeks 4, 8, and 12, and then every 12th week until the end of the study or early termination. Blood samples for analysis of peak eculizumab concentration (C_max_), free C5 concentrations, and hemolytic activity were taken at least 60 min after the completion of eculizumab infusion at Day 1, Weeks 4, 8, and 12, and then every 12th week.

CSF samples (baseline and trough only) for measurement of eculizumab and free C5 concentrations were available from those patients who consented to lumbar puncture procedures and were taken at baseline, Weeks 12 and 24, and then every 24th week until the end of the study or early termination.

Blood samples for measurement of anti-drug antibodies (ADAs) and neutralizing antibodies (NAbs) were taken 5–90 min before infusion of eculizumab at baseline, Weeks 4 and 12, and then every 12th week until the end of the study or early termination.

### Sample Analyses

Analyses of eculizumab and free C5 in serum and CSF, and of *in vitro* hemolytic activity were conducted by Charles River Laboratories (Mattawan, MI, USA) using laboratory-developed tests, validated according to the contemporary recommendations of the US Food and Drug Administration (34) and the European Medicines Agency (35).

#### Pharmacokinetics of Eculizumab in Serum and CSF

Total (bound plus free) concentrations of eculizumab in serum and CSF (where samples were available) were measured by enzyme-linked immunosorbent assays with quantification ranges of 9.38–600 μg/ml and 0.00309–0.400 μg/ml, respectively. Serum analysis used absorbance detection, while CSF analysis used electrochemiluminescence [Meso Scale Discovery (MSD) platform; Meso Scale Diagnostics, Rockville, MD, USA].

#### Anti-AQP4 Antibody Titer

Anti-AQP4 autoantibody titers were measured using a fluorescence-activated cell sorting assay (Mayo Clinic Laboratories, Rochester, MN, USA).

#### Free C5 Concentration

Concentrations of free C5 in serum (as a measure of target engagement) and in CSF (to determine CNS penetration) were measured by ligand-binding assays using eculizumab for free C5 capture and chemiluminescent detection (MSD platform). Quantification ranges in serum and CSF were 0.0274–20 μg/ml and 3.00–400 ng/ml, respectively. Serum free C5 <0.5 μg/ml represents complete inhibition of terminal complement (Alexion Pharmaceuticals, data on file).

#### *In vitro* Hemolytic Activity

Complement-mediated hemolytic activity of patient serum was measured *in vitro* by lysis of chicken red blood cells (cRBCs) using a semi-quantitative assay. cRBCs were sensitized with an anti-cRBC polyclonal antibody, incubated with serum, centrifuged and the supernatant measured using absorbance (415 nm) for hemoglobin released *via* cell lysis. Percent hemolysis was expressed relative to pooled normal human serum, as proof of the pharmacology of eculizumab. cRBC hemolysis <20% represents complete terminal complement inhibition (Alexion Pharmaceuticals, data on file).

#### Immunogenicity

To investigate the presence or absence of an immune response to eculizumab, serum concentrations of ADAs against eculizumab were measured using a solution-phase bridging assay with electrochemiluminescent detection (MSD platform). The method used a tiered approach, with screening (sensitivity 6.26 ng/ml) and confirmatory competition steps to identify samples as positive or negative for the presence of ADAs to eculizumab. If ADAs to eculizumab were detected, the positive ADA samples were analyzed further for titer, and for the presence of NAbs *via* a ligand-binding assay for inhibition of C5 binding with electrochemiluminescent detection (MSD platform).

### Population-Pharmacokinetic Methods

A previously developed model of eculizumab population pharmacokinetics in patients with generalized myasthenia gravis (gMG) (36) was used as the base model for full evaluation of the impact of covariates on eculizumab pharmacokinetics in patients with AQP4-IgG-positive NMOSD. As described in the accompanying paper (36), a two-compartment model with first-order elimination was developed to describe the pharmacokinetic profile of eculizumab in patients with gMG, using serum eculizumab concentration data from the REGAIN study (37). In that model, random effects were included for central clearance (CL) and central volume of distribution (V_1_). A proportional error was used to describe the residual variability of the study data. All pharmacokinetic parameters were allometrically scaled by baseline body weight. Eculizumab CL was estimated with body weight as a covariate at ~ 0.00749 L/h for a typical patient with gMG weighing 70 kg. Plasmapheresis/plasma exchange (PLEX) events were modeled to account for a temporary increase in eculizumab CL during the PLEX administration period.

Model development for population pharmacokinetics in patients with AQP4-IgG-positive NMOSD was carried out in a stepwise manner using first-order, conditional estimation with interaction. Body weight, centered on 70 kg, was considered a structural factor on CL and intercompartmental clearance (Q) and on V_1_ and peripheral volume of distribution (V_2_) parameters. PLEX was considered a structural factor on CL. Body weight and PLEX factors were included early in model development before full covariate evaluation; therefore, they were not part of the covariate searching procedure. The structural pharmacokinetic model (including body weight) was defined as the base model and served as the starting point for covariate assessment.

*Post-hoc* pharmacokinetic parameter random effects (ETAs) obtained from the base structural model were used in an exploratory analysis to identify potential covariate–pharmacokinetic parameter relationships for the formal covariate search.

In order to assess whether dose alterations would be required in particular patient populations, a number of baseline covariates were evaluated, including demographic and clinical factors (see Supplementary Methods for full list). Covariates were identified using a stepwise covariate modeling procedure (see Supplementary Methods). The criteria that guided development of the model that best described eculizumab disposition are detailed in the Supplementary Methods. Model performance and robustness were evaluated by visual predictive check and non-parametric bootstrap analyses.

### Exploratory Analysis of Free C5 and Hemolytic Activity

Exploratory plots were generated and summary statistics calculated for free C5 and hemolytic activity by clinic visit. Because complete inhibition was achieved in almost all patients who received eculizumab, the data were insufficient for development of pharmacokinetic/pharmacodynamic models for either free C5 or hemolytic activity. The previously established threshold eculizumab concentration to achieve complete terminal complement inhibition [see accompanying paper (36)] was used as a benchmark to assess the relationship between eculizumab exposures and pharmacodynamic endpoints.

### Exposure–Response for Efficacy

The efficacy analysis included all eculizumab-treated patients for whom *post-hoc* pharmacokinetic parameters were available. Kaplan–Meier plots of relapse-free survival were generated for the primary endpoint—time to first adjudicated on-trial relapse. For eculizumab-treated patients, time to first adjudicated on-trial relapse was evaluated with respect to quartiles of eculizumab exposure [eculizumab area under the concentration–time curve within one dosing interval, i.e., 2 weeks, at steady state (AUC_ss_)] values predicted from the population-pharmacokinetic model.

### Exposure–Response for Safety

Treatment-emergent adverse events (AEs) of special interest—defined as infections (meningococcal infections, *Aspergillus* infections, other serious infections, or sepsis), infusion reactions, serious cutaneous adverse reactions, cardiac disorders, and angioedema—and treatment-emergent AEs that occurred in ≥ 5% of eculizumab-treated patients were evaluated by treatment and by eculizumab exposure quartile. Individual eculizumab AUC_ss_ values were derived from *post-hoc* exposure estimates and were used as the exposure metric.

### Subgroup Analysis

Subgroup analyses were undertaken in Asian and Japanese patients.

## Results

A total of 96 patients received eculizumab treatment during the PREVENT study; pharmacokinetic data were available for 95 patients, of whom 37 were Asian and nine were Japanese. The numbers of patients with data available for each analysis are shown in Table 1. Patient characteristics, clinical efficacy, and safety results from the PREVENT study have been reported previously (31). In brief, the mean [standard deviation (SD)] baseline characteristics of the 96 eculizumab-treated patients were: age 43.8 (13.4) years; body weight 68.4 (20.2) kg; body mass index 25.8 (7.0) kg/m^2^; estimated glomerular filtration rate 114.2 (37.9) ml/min/1.73 m^2^; white blood cell count 6.5 (2.2) × 10^9^/L; and albumin 43.1 (3.1) g/L. Of the 96 patients, 46 (48%) were White, 37 (39%) were Asian, nine (9%) were Black/African American, and four (4%) were Other; there were nine patients (9%) of Japanese descent. The mean (SD) annualized relapse rate 24 months before screening was 1.9 (0.9) relapses/year. Fifteen PLEX events were recorded for three patients (four, five, and six events, respectively) treated with eculizumab during the study.

**Table 1:** Numbers of patients with data available for the analyses.

**Analysis**	**Number of patients**
Received eculizumab	96
Population-PK (eculizumab serum concentration)	94^a,b^
Serum free C5	95^a^
Percent cRBC hemolysis	95^a^
CSF eculizumab concentration	4^c^
CSF free C5	4^c^
Population-PK: efficacy	95^a,b^
Population-PK: safety	95^a,b^

*C5, complement component 5; cRBC, chicken red blood cell; CSF, cerebrospinal fluid; PK, pharmacokinetic*.

a *Population-PK, serum free C5, and hemolytic-activity data were not available for one patient because of a local protocol requirement; this patient was excluded from the population-PK analysis but was included in the efficacy and safety exposure–response analyses as post-hoc exposure was predicted by the PK model*.

b *Population-PK data were not available for one patient because of an unusual PK profile; no post-hoc PK parameters were obtained for this patient and, therefore, data for this patient were also excluded from the efficacy and safety exposure–response analyses*.

c *Eight eculizumab-treated patients provided informed consent for CSF sampling; however, CSF samples at scheduled visits after the first dose were only available for four eculizumab-treated patients*.

### Pharmacokinetics

#### Serum Eculizumab Concentrations

**Figure 1:**
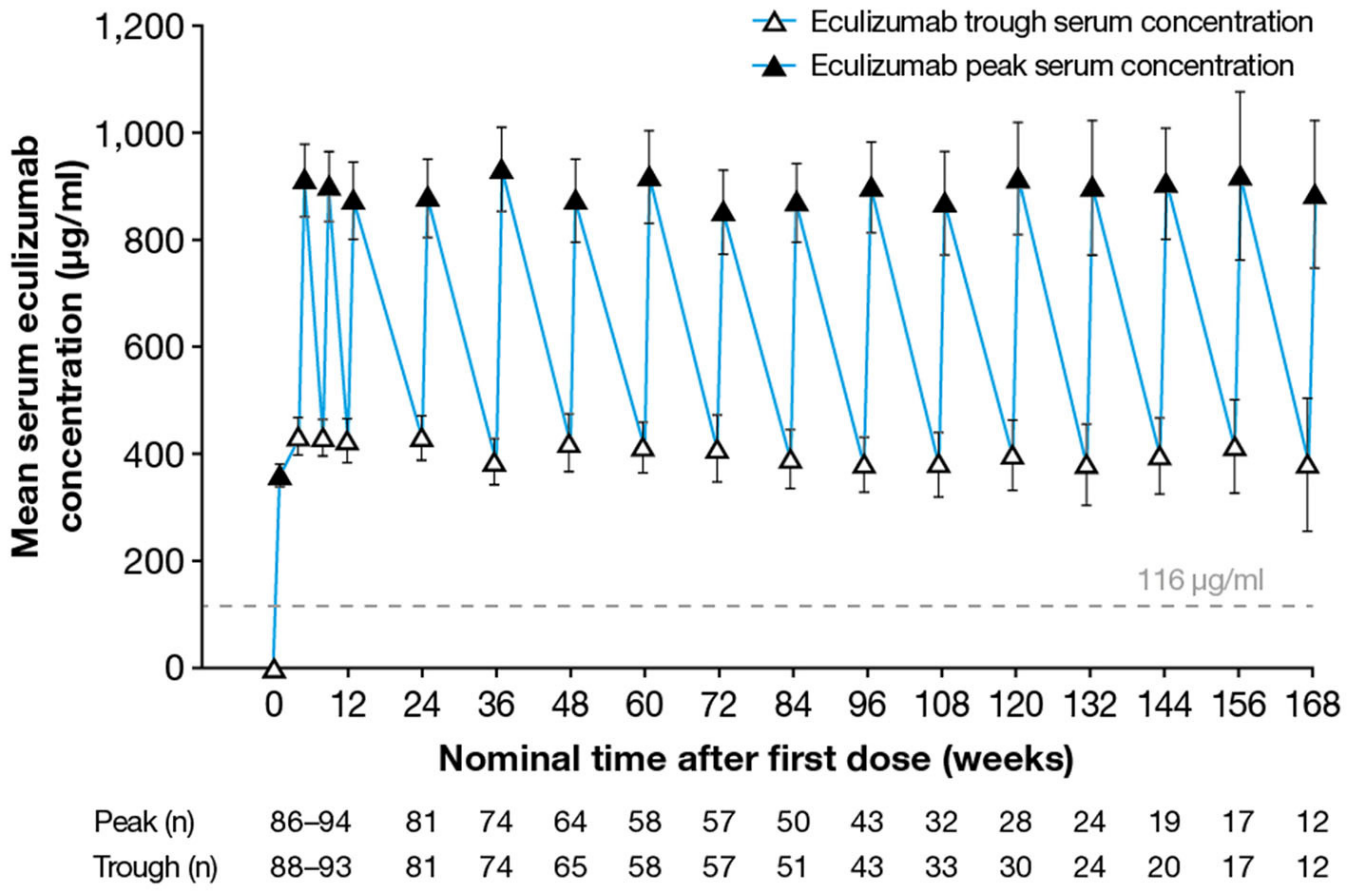
Serum eculizumab concentrations during the study. Mean (95% confidence interval) trough and peak serum eculizumab concentrations in patients who received eculizumab in the PREVENT study; eculizumab concentrations below the lower limit of quantification (9.38 μg/ml) were analyzed as 4.69 μg/ml (half the lower limit of quantification). Eculizumab concentrations above 116 μg/ml (dashed line) indicate sufficient concentration to achieve complete complement inhibition.

Following implementation of the eculizumab 900/1,200 mg dosing regimen, steady-state serum eculizumab concentrations were achieved by Week 4 and were sustained throughout the treatment period. Eculizumab concentrations were above the previously established threshold of 116 μg/ml for complete complement inhibition (36) in 96.7% of all post-baseline samples. At any timepoint studied, 92–100% of patients in whom concentrations were measured had C_trough_ values above the threshold. Serum eculizumab C_trough_ values were maintained well above the 116 μg/ml threshold in all post-baseline samples from the majority of patients (85/95; 89%) (Figure 1). None of the 10 patients who at any time recorded below-threshold C_trough_ concentrations had received PLEX; however, in six of these patients, the blood sample(s) was taken following a delay in the scheduled eculizumab administration (i.e., eculizumab administration, and the pre-dose blood sampling, occurred more than 14 days after the last dose).

Between Weeks 4 and 168, the mean (SD) C_max_ by visit ranged from 850 (297) to 930 (338) μg/ml. Median serum eculizumab C_trough_ was ~ 400 μg/ml, with the majority of individual C_trough_ values ranging from 100 to 700 μg/ml. Between Weeks 4 and 168, the mean (SD) C_trough_ by visit ranged from 378 (167) to 432 (169) μg/ml.

A simulation of the steady-state eculizumab serum concentrations found that eculizumab administration up to 2 days after the scheduled 2-week timepoint maintained C_trough_ values above the 116 μg/ml threshold in almost all patients (data not shown).

#### CSF Eculizumab Concentrations

Post-first dose CSF samples taken at scheduled visits were available for four eculizumab-treated patients. The mean time-matched serum eculizumab concentrations were 402- to 649-fold higher than CSF eculizumab concentrations. In three patients, CSF eculizumab C_trough_ values were close to 1 μg/ml, while the fourth patient showed a CSF C_trough_ value 3- to 4-fold higher than those of the other patients. Within each patient, the CSF C_trough_ values remained stable over time.

#### Population-Pharmacokinetic Model

The two-compartment model with first-order elimination developed for the analysis of eculizumab pharmacokinetics in patients with gMG was used as the base model. However, a number of modifications were implemented. To account for the extended treatment duration in the NMOSD study (data were analyzed for the first 168 weeks of treatment, whereas treatment duration was 26 weeks in the MG study), body weight was included as a time-varying covariate, rather than as baseline body weight. Additionally, an additive effect of PLEX on CL, rather than a multiplicative effect as used in the original model, was found to improve the individual fit of the model for patients undergoing PLEX procedures.

The initial exploration of potential covariate effects showed no trends between any of the covariates of interest and CL or V_1_, except for effects of the baseline serum albumin concentration and the patient's sex on V_1_, which were the only two covariates showing a *p*-value close to 0.01. However, following stepwise forward addition and backward elimination steps to determine the contribution of these covariates, neither met the criteria to be retained in the model.

In the final population-pharmacokinetic model, the eculizumab pharmacokinetic data from patients with NMOSD were well-described by a two-compartment model with first-order elimination. The final model parameter estimates showed good precision with percent standard error of estimation always below 26% and acceptable inter-individual variability (ETA) shrinkage. Parameter estimates for the final population-pharmacokinetic model are provided in Supplementary Table 1. Bayesian estimates of the pharmacokinetic parameters are summarized in Table 2: the mean (SD) terminal half-life was estimated to be 414 (103) h, and the mean (SD) CL was estimated to be 0.00759 (0.00313) L/h.

**Table 2:** Summary of estimated pharmacokinetic parameters.

**Statistic**	**Mean**	**SD**	**Median**	**2.5th percentile**	**97.5th percentile**	**Minimum**	**Maximum**
CL (L/h)	0.00759	0.00313	0.00668	0.00418	0.0154	0.00362	0.0204
V_1_ (L)	2.33	0.527	2.21	1.58	3.4	1.56	3.68
V_2_ (L)	2.11	0.367	2.06	1.56	2.9	1.49	3.09
Q (L/h)	0.238	0.0638	0.226	0.148	0.382	0.138	0.42
C_max, ss_ (μg/ml)	928	241	898	525	1,380	453	1,530
C_trough, ss_ (μg/ml)	405	152	416	140	706	92.7	811
Terminal half-life^a^ (h)	414^b^	103	438	238	641	202	765
AUC_ss_ (μg·h/ml)	180,000	57,900	180,000	77,900	287,000	58,700	331,000
Accumulation ratio	2.06	0.381	2.05	1.32	2.8	1.19	3.28

*Data are summaries of the individual Bayesian estimates*.

a *Terminal half-life was calculated as: (V_1_ + V_2_)/CL ,100 ln(2)*.

b *Terminal half-life mean was calculated as harmonic mean*.

*AUC, area under the concentration–time curve within one dosing interval; CL, clearance; C_max_, peak concentration; C_trough_, concentration at the end of the dosage interval; Q, intercompartmental clearance; SD, standard deviation; ss, steady state; V_1_, volume of distribution in the central compartment; V_2_, volume of distribution in the peripheral compartment*.

Random effects were included for CL and V_1_ and a correlation term was estimated between the two parameters. All pharmacokinetic parameters were allometrically scaled by time-varying body weight; in addition, PLEX events were modeled to account for a temporary increase in eculizumab CL during the PLEX period. Eculizumab pharmacokinetic values were not influenced by any of the other covariates examined.

**Figure 2:**
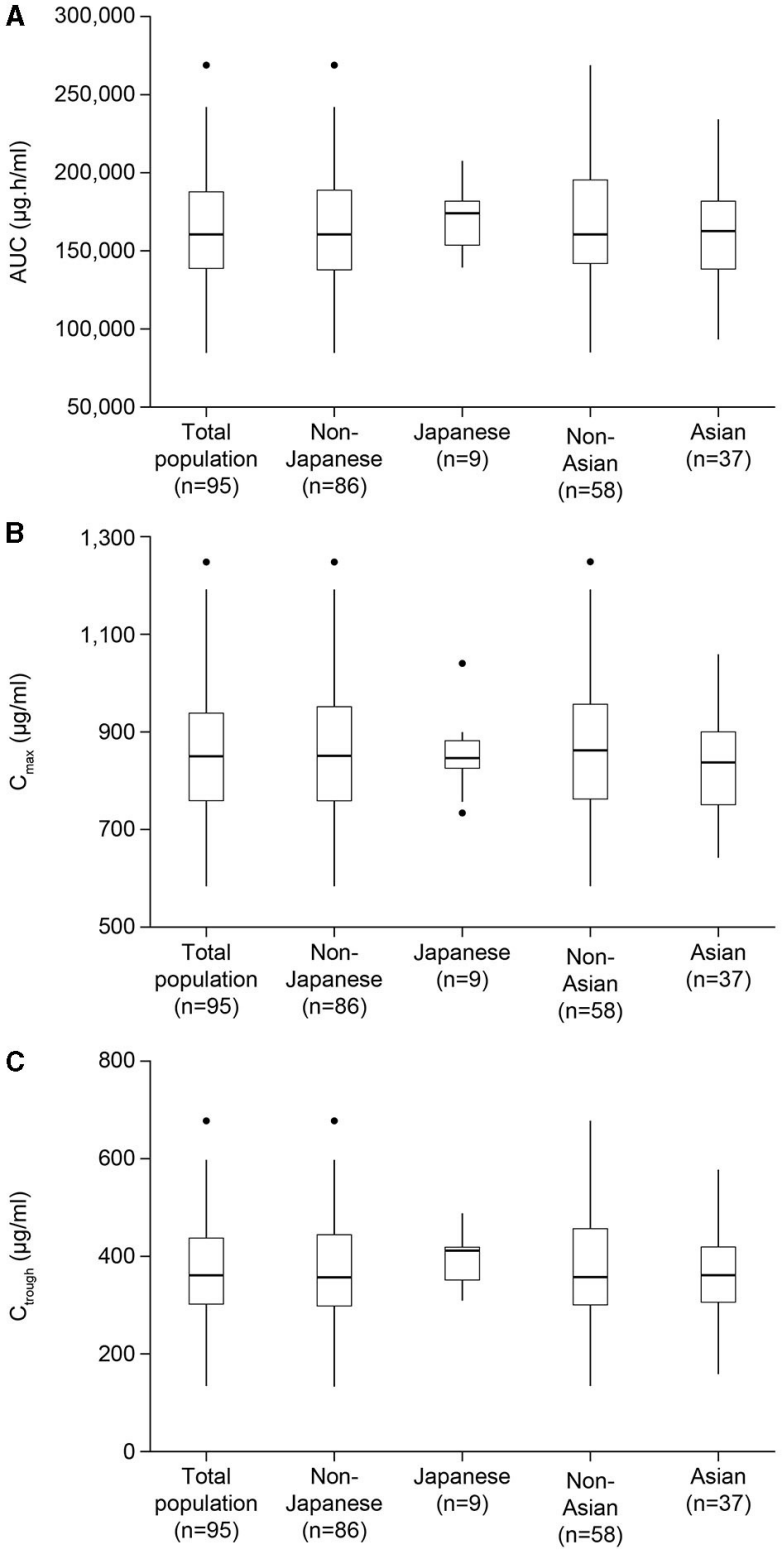
Estimated body weight-normalized eculizumab exposure parameters for the total patient population, Asian, non-Asian, Japanese, and non-Japanese patients. Results are shown for parameters at steady state: **(A)** AUC, **(B)** C_max_, and **(C)** C_trough_. Exposures were normalized by body weight using the exponent estimated in the final model. AUC, area under the concentration–time curve within one dosing interval; C_max_, peak concentration; C_trough_, concentration at the end of the dosage interval; ss, steady state.

No differences were observed in steady-state eculizumab exposure parameters AUC, C_max_, and C_trough_ between Asian and non-Asian patients or between Japanese and non-Japanese patients, after normalization for body weight (Figure 2). Further details of eculizumab pharmacokinetic parameters in Asian and non-Asian patients, and in Japanese and non-Japanese patients are provided in Supplementary Tables 2, 3, respectively.

### Immunogenicity

Anti-eculizumab immunogenicity was negligible, with two ADA-positive samples, one each from two patients. ADA titers for these two patients were low and transient; their subsequent samples were negative on ADA screening. There was no impact on observed pharmacokinetic/pharmacodynamic data in either patient, and neither patient experienced relapse of NMOSD. There were no NAb-positive samples.

### Pharmacodynamics

The eculizumab threshold concentration to achieve complete terminal complement inhibition (116 μg/ml) has been established using population-pharmacokinetic/pharmacodynamic data from the REGAIN study in patients with gMG (36). Given the consistency of eculizumab pharmacokinetics/pharmacodynamics across indications, this threshold was used as a benchmark to assess the relationship between eculizumab exposure and the pharmacodynamic endpoint.

#### Serum Free C5 Concentrations

**Figure 3:**
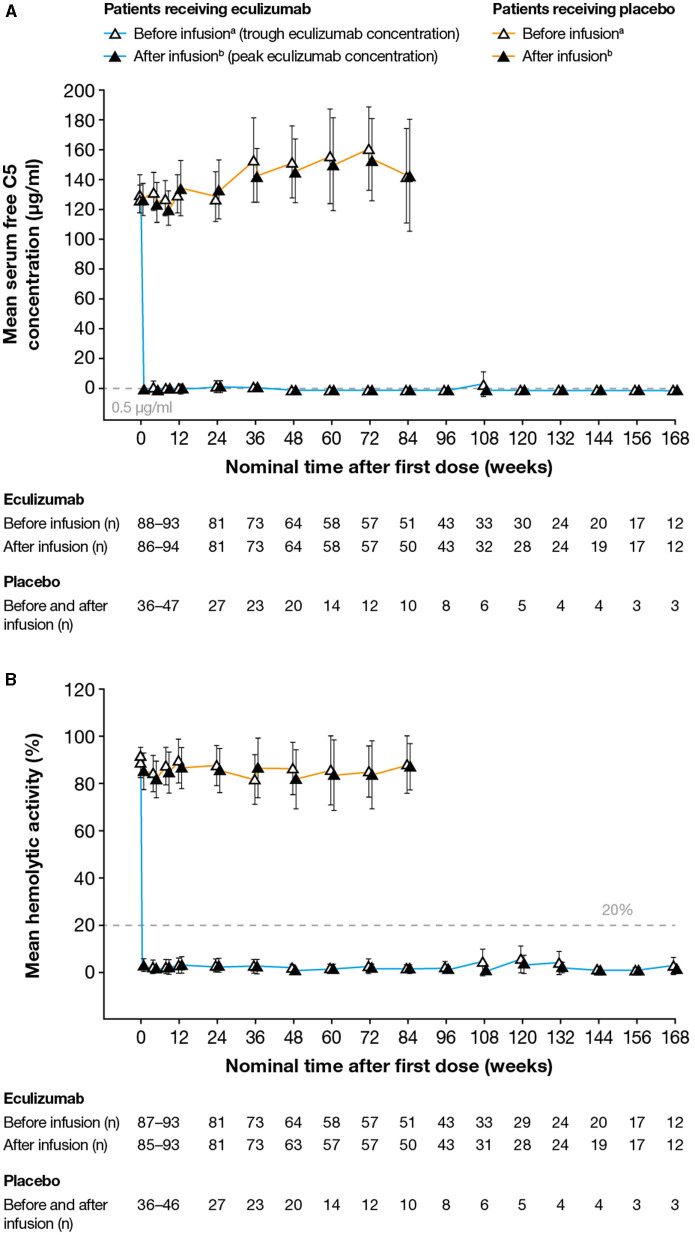
Serum free C5 concentrations and complement-mediated hemolytic activity in serum during the study. **(A)** Mean (95% CI) serum free C5 concentrations. Free C5 concentrations below the lower limit of quantification (0.0274 μg/ml) were analyzed as 0.0137 μg/ml (half the lower limit of quantification). Free C5 concentrations below 0.5 μg/ml (dashed line) indicate complete terminal complement inhibition. No free C5 data were available for one patient. **(B)** Mean (95% CI) percent *in vitro* complement-mediated hemolytic activity of serum samples. Hemolysis values above 20% (dashed line) indicate incomplete inhibition of hemolysis. No hemolysis data were available for one patient. For both analyses, samples were taken before and after eculizumab infusion (i.e., at eculizumab serum trough and peak concentrations, respectively); samples taken before and after infusion in patients receiving placebo are shown for comparison. C5, complement protein 5; CI, confidence interval. ^a^Samples taken 5–90 min before infusion. ^b^Samples taken 60 min after the completion of infusion. Data are plotted for timepoints when samples were available for ≥ 10 patients. The numbers reported below the graphs are the numbers of patients for whom samples were tested at that timepoint.

**Figure 4:**
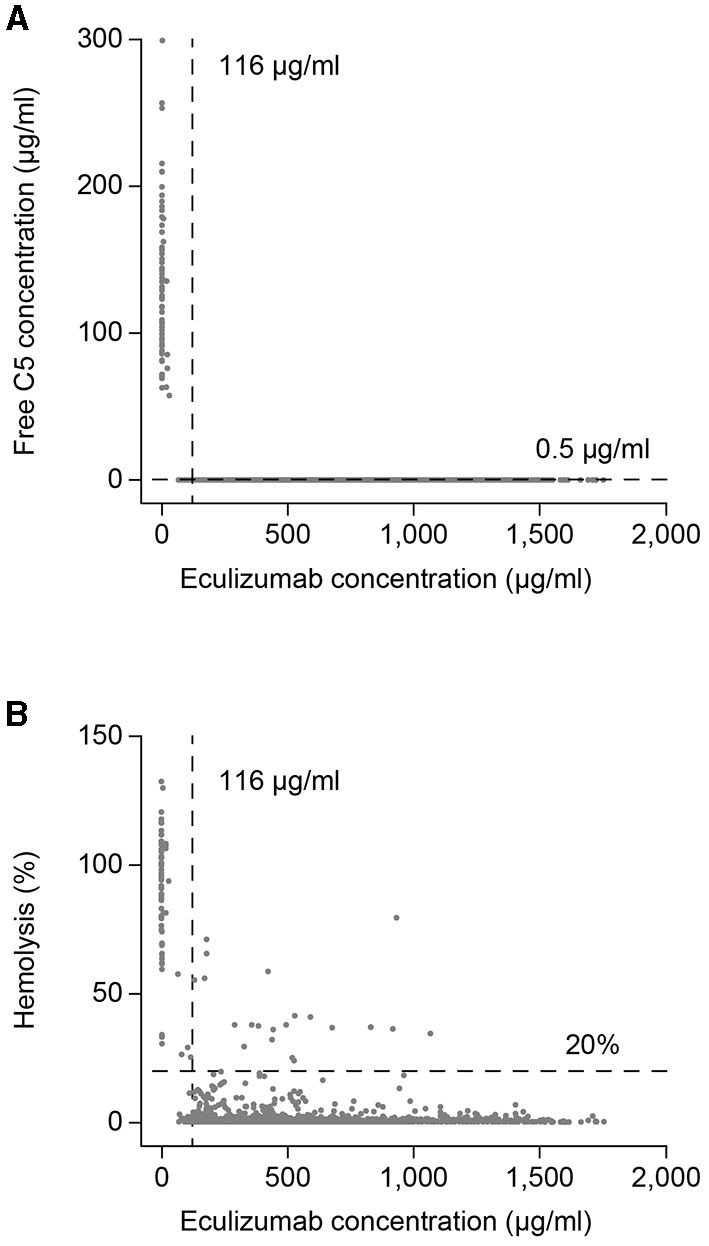
Eculizumab exposure–response profiles for serum free C5 concentration and hemolytic activity. Scatterplots of **(A)** time-matched serum free C5 concentrations and eculizumab concentrations, and **(B)** time-matched percent hemolysis and eculizumab concentrations. Vertical lines at eculizumab 116 μg/ml and horizontal lines at free C5 0.5 μg/ml in panel A and 20% hemolysis in panel B show thresholds representing complete terminal complement inhibition for serum eculizumab concentration, and for free C5 concentration and hemolytic activity, respectively. C5, complement component 5.

At any post-baseline timepoint studied, 97–100% of patients in whom concentrations were measured had free C5 concentrations <0.5 μg/ml. Serum free C5 concentrations <0.5 μg/ml were observed in 99.3% of post-baseline samples from eculizumab-treated subjects, and in all post-baseline samples (observed at Day 1 eculizumab C_max_ and at all visits at the time of eculizumab C_trough_) from 91/95 (96%) of eculizumab-treated patients (Figure 3A). In the remaining patients, serum free C5 concentrations <0.5 μg/ml were seen at the time of C_trough_ at the majority of visits during the maintenance phase of treatment, except for one patient who consistently showed serum free C5 concentrations > 0.5 μg/ml for all samples available up to Week 36. The relationship between eculizumab exposure and free C5 concentration confirms that at the exposure range achieved in the study, serum free C5 concentrations were reduced below the 0.5 μg/ml threshold for complete inhibition of terminal complement (Figure 4A).

#### CSF Free C5 Concentrations

Pre-first-dose CSF samples were available for eight eculizumab-treated patients (median free C5 concentration 253 ng/ml, SD 108 ng/ml). Post-first-dose CSF samples at scheduled visits were available for four eculizumab-treated patients. All post-first-dose CSF free C5 concentrations were below the lower limit of quantification of 3.00 ng/ml, indicating complete terminal complement inhibition in CSF.

#### Hemolysis

At any timepoint studied, 90–100% of patients in whom concentrations were measured demonstrated complete inhibition of terminal complement, as defined by <20% *in vitro* cRBC hemolysis. Overall, 97.7% of all post-baseline samples achieved *in vitro* cRBC hemolysis <20%. Additionally, *in vitro* cRBC hemolysis <20% was achieved at all time points (Day 1 eculizumab C_max_ and at all visits at the time of eculizumab C_trough_) in nearly all treated patients (88/95; 93%) (Figure 3B). The relationship between eculizumab exposure and hemolytic activity confirms that at the achieved eculizumab exposure range, hemolytic activity was reduced below the threshold of 20% for the large majority of assessments (Figure 4B). In a few samples (~ 1.9% of the total) hemolytic activity was determined to be ≥ 20% when serum eculizumab concentration was ≥ 116 μg/ml; this observation is likely to reflect assay variability.

### Exploratory Exposure–Response Analysis

#### Time to First Adjudicated On-Trial Relapse

**Figure 5:**
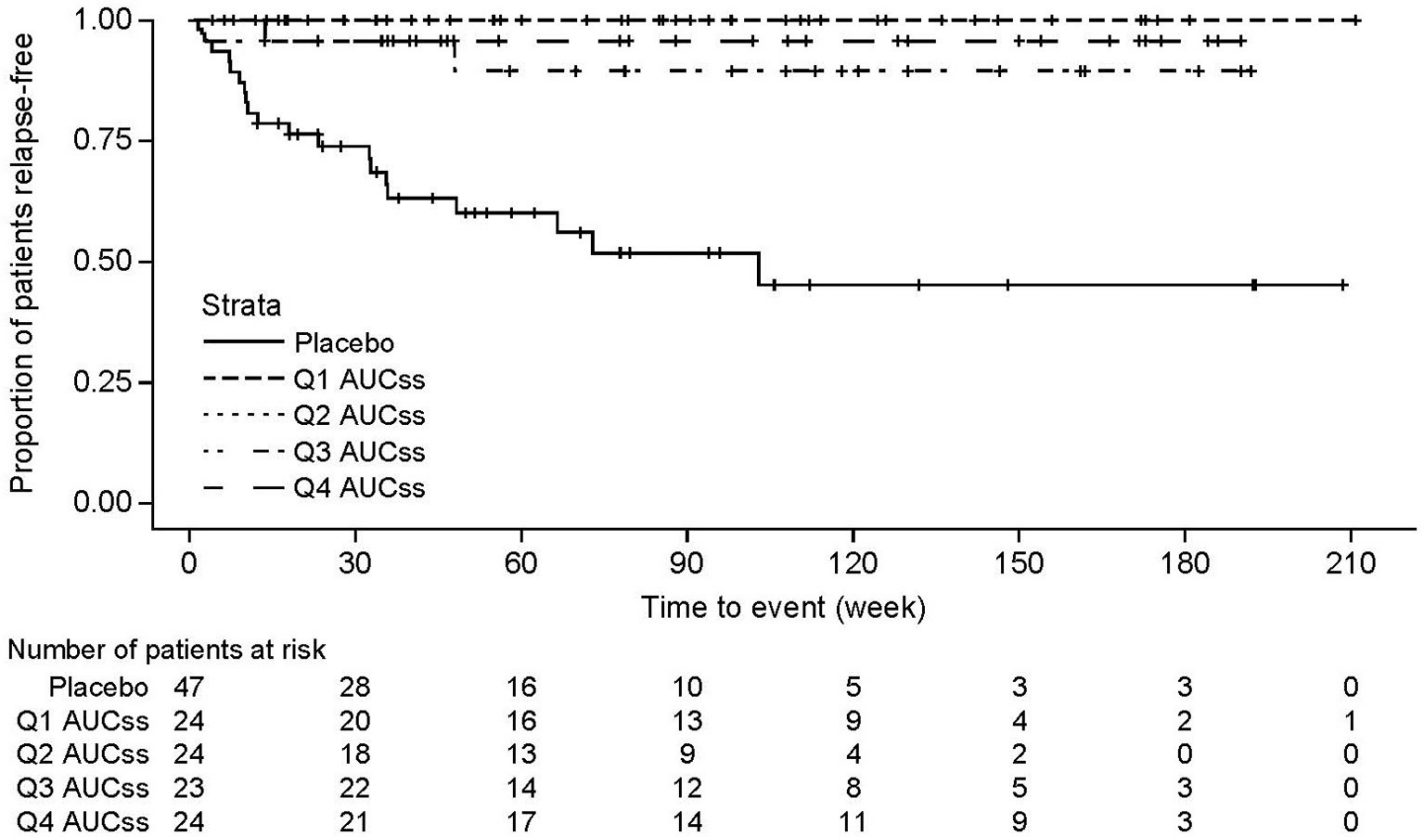
Kaplan–Meier survival plots for time to first adjudicated on-trial relapse, according to eculizumab exposure. Relapse-free survival curves for each exposure quartile for time to first adjudicated on-trial relapse in eculizumab-treated patients. For comparison, the survival curve is also shown for patients receiving placebo. AUC_ss_ is based on the maintenance dose of 1,200 mg eculizumab. One eculizumab-treated patient for whom no *post-hoc* pharmacokinetic parameters were obtained was excluded from the analysis. AUC_ss_, area under the concentration–time curve within one dosing interval at steady state; Q1, 1st quartile; Q2, 2nd quartile; Q3, 3rd quartile; Q4, 4th quartile.

Efficacy results have been reported previously (31). Adjudicated on-trial relapses occurred in 3/96 of eculizumab-treated patients (3%) (Table 3). Complete complement suppression was achieved, based on serum free C5 and hemolysis data, in each of these three patients. Kaplan–Meier survival plots of time to first adjudicated on-trial relapse showed the treatment effect of the eculizumab 900/1,200 mg dose regimen. Consistent with the significant effect of eculizumab dosing on relapses, there was a clear separation in relapse-free survival across all exposure quartiles for eculizumab patients compared with patients in the placebo group. Furthermore, it can be seen that there was no clear separation between the relapse-free survival curves across the different exposure quartiles, suggesting a maximal treatment effect was achieved within the observed exposure range (Figure 5). These findings were supported by data for on-trial relapses as determined by the treating physician (see Supplementary Material).

**Table 3:** Number of patients with adjudicated on-trial relapse, by eculizumab exposure quartile.

**Group**	**Total no. of patients**	**No. of patients with adjudicated on-trial relapse**
Patients receiving placebo	47	20
**Patients receiving eculizumab**		
Patients with AUC_ss_ within 1st quartile (range 58,714–143,644 μg·h/ml)	24	0
Patients with AUC_ss_ within 2nd quartile (range 144,679–179,692 μg·h/ml)	24	0
Patients with AUC_ss_ within 3rd quartile (range 181,113–216,888 μg·h/ml)	23	2
Patients with AUC_ss_ within 4th quartile (range 218,938–331,373 μg·h/ml)	24	1

*AUC_ss_ was calculated using post-hoc pharmacokinetic parameters from the final population-pharmacokinetic model. One eculizumab-treated patient for whom no post-hoc pharmacokinetic parameters were obtained was excluded from the analysis*.

*AUC, area under the concentration–time curve within one dosing interval; ss, steady state*.

#### Impact of Eculizumab Exposure on Anti-AQP4 Antibodies

Eculizumab serum concentrations were similar across quartiles of anti-AQP4 antibody titers, consistent with the expectation that eculizumab concentrations would not impact anti-AQP4 autoantibody titers.

#### Safety

No trends were observed in AEs or AEs of special interest with increasing eculizumab exposure, comparing patients treated with eculizumab stratified in terms of the steady-state AUC quartiles (see Supplementary Table 5). Similarly, no meaningful safety trends were observed when comparing Asian vs. non-Asian patients and Japanese vs. non-Japanese patients. No differences were observed in AEs or AEs of special interest between eculizumab-treated patients and those who received placebo.

## Discussion

The results of this pharmacokinetic/pharmacodynamic analysis in patients with AQP4-IgG-positive NMOSD endorse the recommended dosing regimen for eculizumab (900 mg weekly for the first four doses, followed by 1,200 mg 1 week later and every 2 weeks thereafter). This analysis also verifies the association between eculizumab's mechanism of action and the efficacy demonstrated in clinical studies (31, 38).

Pharmacokinetic data were well-described by a two-compartment model with first-order elimination, based on a model developed previously to describe the pharmacokinetic profile of eculizumab in patients with gMG (36). It is of note that the pharmacokinetic model parameters are similar for patients with NMOSD and those with gMG; the typical CL and terminal half-life values of eculizumab for a person weighing 70 kg are almost identical in the two clinical conditions.

Sufficient serum concentrations of eculizumab were achieved from Day 1 of treatment, with complete terminal complement inhibition observed by the end of infusion of the first dose, and sustained throughout the entire treatment period in nearly all patients (serum free C5 <0.5 μg/ml in 96% and *in vitro* cRBC hemolytic activity <20% in 93%). The rapid and sustained C5 suppression following eculizumab administration accounts for the significant treatment effect reported for the primary endpoint (first adjudicated on-trial relapse) and reduction in relapse risk observed in PREVENT (31). Complete complement suppression was achieved (based on serum free C5 concentration) in the three patients with adjudicated on-trial relapses who were treated with eculizumab, suggesting that other factor(s) may have triggered the relapse. It should be noted that complete terminal complement inhibition by eculizumab significantly reduced the risk of adjudicated relapse compared with placebo [hazard ratio 0.06 (95% CI: 0.02, 0.20); *p* < 0.001] (31).

The ~ 17-day terminal elimination half-life of eculizumab in patients with NMOSD allows a convenient interval of 2 weeks between doses, providing a small amount of leeway in dosing (± 2 days) without risking incomplete complement inhibition (free C5 > 0.5 μg/ml). Consistent with the prescribing information to administer the maintenance dose of eculizumab within 2 days of the 2-weekly recommended dosing timepoint in patients with NMOSD, simulation of the steady-state eculizumab serum concentrations suggests that administration within this time interval would maintain C_trough_ values above the 116 μg/ml threshold in almost all patients. Day-to-day fluctuations in C5 concentrations are possible as a result of complement-activating events such as infection, surgery, and pregnancy; the eculizumab dosing regimen helps prevent C5 concentration increases translating into clinical relapse.

This analysis showed that in 89% of patients with AQP4-IgG-positive NMOSD, the 2-week dosing interval provided sustained minimum serum eculizumab concentrations above the threshold (116 μg/ml) necessary to achieve complete terminal complement inhibition [as identified by exposure–response modeling in patients with gMG (36)]. In the remaining 12% of patients, the samples at the majority of timepoints showed eculizumab concentrations > 116 μg/ml. Overall, 96.7% of the post-dose samples showed eculizumab concentrations > 116 μg/ml. In addition, clinical efficacy was consistent across the range of steady-state serum eculizumab concentrations achieved in individual patients, confirming that the recommended dose has been optimized. This is consistent with the pharmacokinetic results from the Phase 3 study in patients with gMG (36).

The model confirmed that PLEX increased eculizumab clearance, which would require a temporary increase in dose during the plasma exchange period (39); fresh frozen plasma infusions are also likely to necessitate an eculizumab dose alteration due to the presence of complement proteins. Current recommendations (22, 23) are to administer a supplemental dose of eculizumab within 60 min after the end of each plasmapheresis/PLEX session, and 60 min before each infusion of fresh frozen plasma.

Although some pharmacokinetic parameters were affected by body weight, the data suggest that no dose adjustments are needed in overweight patients. None of the other patient factors tested in the model significantly affected eculizumab pharmacokinetics, suggesting that dose adjustments in specific populations (for example in patients with renal/hepatic impairment) are unnecessary to maintain a favorable benefit–risk profile in patients with NMOSD.

CSF free C5 concentrations were below the lowest measurable concentration (3.00 ng/ml) after the first dose of eculizumab in all four patients in PREVENT with available data. This supports the findings of an earlier Phase 2 study in patients with AQP4-IgG-positive NMOSD in which eculizumab was shown to penetrate the CNS, with a mean (SD) CSF concentration of 34.7 (18.7) ng/ml in 11 patients at 3 months of treatment (38). CSF free C5 concentrations were significantly reduced at 3 months, from a mean (SD) of 144 (75.5) ng/ml in 12 patients at screening to 60.8 (23.3) ng/ml in five of the 11 patients who were still receiving eculizumab at 3 months and undetectable in the other six patients (38). At present it is unknown whether free C5 suppression observed in CSF is a surrogate for complement suppression in other brain compartments where C5 activation may be involved in inducing relapses.

There was no apparent relationship between eculizumab serum concentration and anti-AQP4 antibody titer, which was to be expected as eculizumab's mechanism of action is distinct from interaction with anti-AQP4 antibodies.

There was a very low incidence in the PREVENT study population of samples testing positive for ADAs with eculizumab and no NAbs were observed. Only two patients transiently tested positive for ADA, each in one sample, both of which were negative for NAbs. This is consistent with studies of eculizumab in paroxysmal nocturnal hemoglobinuria (PNH), atypical hemolytic uremic syndrome, and gMG (36, 40, 41). Reducing the potential for immunogenicity was a key consideration when engineering the eculizumab molecule and was achieved by grafting the murine complementarity-determining regions into human antibody frameworks (29). The absence of NAbs indicates that sustained efficacy with long-term use of eculizumab can be expected (42).

In the PREVENT study, the incidence of AEs considered related to treatment was similar in the eculizumab and placebo groups, as was the incidence of serious AEs; most AEs were mild to moderate in severity (31). The current analysis showed no safety trends regarding AEs or AEs of special interest (infections, infusion reactions, serious cutaneous adverse reactions, cardiac disorders, and angioedema) with increasing eculizumab exposure as stratified by steady-state AUC quartiles. The membrane attack complex plays a key role in clearing *Neisseria meningitidis* bacteria and so blocking this pathway with eculizumab may increase the risk of meningococcal disease (23). All patients were vaccinated against *N. meningitidis* before participating in the study, and no cases of meningococcal infection were reported during the study. *Neisseria meningitidis* serogroups ACWY vaccination and, where available, *N. meningitidis* serogroup B vaccination, are strongly recommended before initiating eculizumab therapy (22, 23). In an analysis of 28,518 person-years (PY) of exposure to eculizumab in the treatment of PNH and atypical hemolytic uremic syndrome, accumulated over almost 10 years of post-marketing pharmacovigilance surveillance worldwide, the overall incidence of meningococcal disease was found to be 0.25/100 PY (43). As seen in the general population following introduction of the Men ACWY meningococcal vaccine in 2005, the rate of meningococcal infection in this eculizumab-treated patient population decreased over time from 0.57/100 PY in 2007 to 0.16/100 PY in 2016 (43).

Data on drug–drug interactions are limited, but the model used in this study incorporated concomitant medications as covariates, including systemic antibacterials, immunosuppressants, antihypertensives, and steroids. None of these medications impacted the pharmacokinetic profile of eculizumab, suggesting a lack of interaction with eculizumab. This is expected for a monoclonal antibody such as eculizumab, which is unlikely to be subject to cytochrome P450 or other enzymatic metabolism.

No differences were observed in the present analysis between Asian/non-Asian and Japanese/non-Japanese patients with regard to pharmacokinetic parameters, complete complement inhibition, or safety outcomes analyzed by eculizumab exposure.

Study limitations for this analysis include the fact that CSF data were limited owing to a very low rate of patients consenting to undergo lumbar puncture; interpretation of the CSF data should therefore be treated with caution. Data on CSF concentrations of markers and drugs are often lacking due to the invasive procedure required to collect samples; however, these (limited) CSF data from PREVENT confirm the Phase 2 study data (38). A second limitation is that only a low proportion of patients in the current analysis were Black/African American, restricting the possibility of a meaningful comparison between ethnic groups.

In summary, this rigorous quantitative assessment of the pharmacokinetic and pharmacodynamic data confirms the recommended dose regimen of eculizumab (900/1,200 mg) has a favorable benefit–risk profile with significant clinical efficacy in patients with AQP4-IgG-positive NMOSD, without the need for dose adjustments in specific populations. Eculizumab was shown to provide rapid, complete, sustained, and well-tolerated inhibition of terminal complement activation in these patients, which translates to the significant reduction of NMOSD relapses.

## Data Availability Statement

The datasets presented in this article are not readily available; Alexion will consider requests for disclosure of clinical study participant-level data provided that participant privacy is assured through methods like data de-identification, pseudonymization, or anonymization (as required by applicable law), and if such disclosure was included in the relevant study informed consent form or similar documentation. Qualified academic investigators may request participant-level clinical data and supporting documents (statistical analysis plan and protocol) pertaining to Alexion-sponsored studies. Further details regarding data availability and instructions for requesting information are available in the Alexion Clinical Trials Disclosure and Transparency Policy at https://alexion.com/our-research/research-and-development. Requests to access the datasets should be directed to https://alexion.com/contact-alexion/medical-information.

## Ethics Statement

The studies involving human participants were reviewed and approved by Independent Ethics Committees or Institutional Review Boards at the 70 centers that participated in the study (see Supplementary Material for full list). The patients/participants provided their written informed consent to participate in this study.

## Author Contributions

PS: analysis planning and strategy, data analyses and interpretation, report writing, critical review, editing, and approval of manuscript. XG: protocol design, analysis planning and strategy, data analyses and interpretation, report writing, critical review, editing, and approval of manuscript. HJK: data analysis, interpretation, report writing, critical review, editing, and approval of manuscript. FB: data analysis, interpretation, critical review, editing, and approval of manuscript. RP: bioanalyses, critical review, editing, and approval of manuscript. All authors contributed to the article and approved the submitted version.

## Funding

Editorial assistance was provided by Duncan Porter and Jackie Mayne of Piper Medical Communications, funded by Alexion Pharmaceuticals Inc. The authors declare that this study received funding from Alexion Pharmaceuticals Inc. The funder had the following involvement with the study as sponsor: study design; collection, analysis, interpretation of data; the writing of this article; and the decision to submit it for publication.

## Conflict of Interest

PS and XG were employees of Alexion Pharmaceuticals, Inc. at the time the work described in this paper was undertaken; RP is an employee of Alexion Pharmaceuticals, Inc.; HJK and FB are employees of Certara Strategic Consulting, which received funding from Alexion Pharmaceuticals. The authors declare that this study received funding from Alexion Pharmaceuticals Inc. The funder had the following involvement with the study as sponsor: study design; collection, analysis, interpretation of data, the writing of this article, and the decision to submit it for publication. Editorial assistance was provided by Piper Medical Communications, funded by Alexion Pharmaceuticals Inc.

## Publisher's Note

All claims expressed in this article are solely those of the authors and do not necessarily represent those of their affiliated organizations, or those of the publisher, the editors and the reviewers. Any product that may be evaluated in this article, or claim that may be made by its manufacturer, is not guaranteed or endorsed by the publisher.
