# Supplementary material for: Eculizumab Pharmacokinetics and Pharmacodynamics in Patients With Neuromyelitis Optica Spectrum Disorder
Source: Front Neurol. 2021 Nov 3;12:696387. doi: 10.3389/fneur.2021.696387 (PMC8597263; doi:10.3389/fneur.2021.696387)
Supplement: Supplementary file 1 [file Data_Sheet_1.docx]

Supplementary Material

# Supplementary Methods

## Software

Data exploration and supplemental data management were conducted using R 3.1.1 (1) and SAS (version 9.4, SAS Institute Inc., Cary NC, USA).

The population-pharmacokinetic analysis was performed using NONMEM (version 7.3) (2) and Perl-speaks-NONMEM (PsN; version 4.2.0). Post processing of the data was conducted using R 3.1.1.

The exploratory pharmacokinetic/pharmacodynamic analyses for hemolytic activity and free C5 concentration and exposure–response analysis for efficacy endpoints were conducted using R 3.1.1.

## Pharmacokinetic Model Development

### Covariates

In order to assess whether dose alterations would be required in particular patient populations, the following baseline covariates were evaluated: demographic factors [age, body weight, height, body mass index, sex, race, and ethnicity (Japanese descent)]; baseline estimated glomerular filtration rate and serum creatinine concentration; baseline measurements of alanine transaminase, aspartate transaminase, alkaline phosphatase, albumin, and bilirubin concentrations and lymphocyte count; baseline anti-aquaporin-4 antibody titer; baseline historical annualized relapse rate in the last 24 months; Expanded Disability Status Scale stratum at baseline; use and type of immunosuppressant therapy at baseline; geographic region; use of anti-anemic, systemic antibacterial, antihypertensive, or anti-infective medications.

For baseline covariates, *post-hoc* pharmacokinetic parameter random effects [inter-individual variability (ETAs)] obtained from the base structural model were used in an exploratory analysis to identify potential covariate–pharmacokinetic parameter relationships to apply in the formal covariate search.

Exploratory techniques included:

- Scatterplots of ETA vs. continuous covariates with locally weighted scatterplot smoothing lines and corresponding *p*-value.
- Box plots of ETA vs. categorical covariates with analysis of variance *p*-value.

Selection of the covariate relationships to test on the population-pharmacokinetic model was based on the results of the exploratory evaluation (*p*-value close to 0.01, threshold for forward inclusion in the formal stepwise covariate modeling procedure analysis).

An automated forward inclusion followed by backward elimination procedure was applied to selected covariates using the stepwise covariate modeling tool as implemented in PsN. In the forward step, covariates were added to the model one by one and retained if they met the retention criteria. Next, each covariate effect retained during the forward step was eliminated from the model in a stepwise manner, until removal of any remaining covariate resulted in a meaningful degradation in the model fit (3).

The model resulting from the backward elimination step, including any remaining covariates, was considered as the final population-pharmacokinetic model of the analysis described in this report.

The effects of continuous covariates, such as age, on model parameters were initially tested with a linear slope function centered on the median covariate value as follows:

*Equation 1*

If a significant parameter–covariate relationship was identified, a power function was evaluated for potential to further improve the model fit as follows:

*Equation 2*

In these relationships, *P*_i_ is the estimated parameter value for individual i, *P_TV_* is the typical value for the parameter, θ is the estimated extent of the effect, cov_i_ is the covariate for the ith individual, cov-tilde is the median covariate value, and η_i_ (ETA) is the deviation of the ith individual from the typical value mean.

The effects of categorical covariates, such as sex, on model parameters were included as follows:

*Equation 3*

In which θ_cov,c_ is the fractional change in P_TV_ per category of covariate cov, and I_cov,c,i_ is an indicator variable, having a value 0 for the most common category and a value of 1 for each additional category.

### Criteria for Covariate Assessment

After graphical exploration of the covariates as described above, the selected covariates were tested using a formal statistical approach, i.e., stepwise covariate modeling, which involved stepwise testing of covariate relationships in a forward inclusion [change in objective function value (ΔOFV) of 6.63; *p* < 0.01 for 1 degree of freedom (DF)] and backward exclusion (ΔOFV of 10.83; *p* < 0.001 for 1 DF) process. For categorical covariates, the ΔOFV for the respective *p*-values could be different depending on the DFs.

### Criteria for Model Acceptance

Selection of the model that best described eculizumab disposition was guided by a number of criteria. These included successful numerical convergence; a successful covariance step; an acceptably low condition number; a decrease in OFV; goodness-of-fit plots with good agreement of model predictions and observed data; an acceptable visual predictive check (VPC); acceptable precision of estimated parameters (preferably a relative standard error of estimation no higher than 50% of the parameter estimate) with physiologically plausible estimated values; and no significant bias in the random effects estimates.

Model development was conducted in a stepwise manner. For nested models, the level of significance for retention of an additional model parameter compared with a model without that parameter was *p* < 0.01 by the likelihood ratio test, corresponding to a drop in OFV of 6.63 points.

### Diagnostic Plots

Within-subject variability diagnostic plots of observed data (dependent variable) vs. population (PRED) and individual predictions (IPRED) were examined for adequate fit. Plots of conditional weighted residuals (CWRES) vs. PRED and vs. the independent variable were inspected for evidence of systematic lack of fit and to confirm the absence of bias in the error distributions. The standard normal distribution of CWRES – a requirement for an unbiased model fit – was checked using the quantile-quantile normal plot.

The ETA deviations from the population mean are expected to be normally distributed with mean zero. To verify absence of bias, scatterplots of between-subject random effects were drawn vs. key continuous model covariates with potential trends visualized with a locally weighted scatterplot smoothing estimator (LOESS) smoothed line, and in a box-and-whisker plot vs. key categorical model covariates.

### Model Performance and Robustness

#### Visual predictive check

VPCs were performed using the VPC tool as implemented in the PsN software package. The mean and 90% prediction interval were plotted, and the observed data were overlaid with observed median and 5th and 95th percentiles (Supplementary Figure 1). To evaluate the model performance across the observed population, VPCs were stratified by key covariates. Essentially complete overlap of simulated and observed quantiles qualifies a model as suitable to predict the data.

#### Nonparametric bootstrap analysis

A bootstrap procedure was carried out to obtain parameter uncertainty estimates and to identify influential observations. In the bootstrap resampling technique, bootstrap replicates were generated by sampling randomly from the original data set with replacement. One thousand replicate data sets were obtained using the bootstrap option in PsN. This resampling was stratified based on treatment and key covariates. Parameter estimates for each of the resampled data sets were obtained by fitting the final model using NONMEM. Nonparametric confidence intervals of the parameter estimates were derived. Bootstrap estimates of the fixed and random effects should be in good agreement with final model estimates.

**SUPPLEMENTARY FIGURE 1 |** Prediction-corrected visual predictive check of final model (maintenance phase).


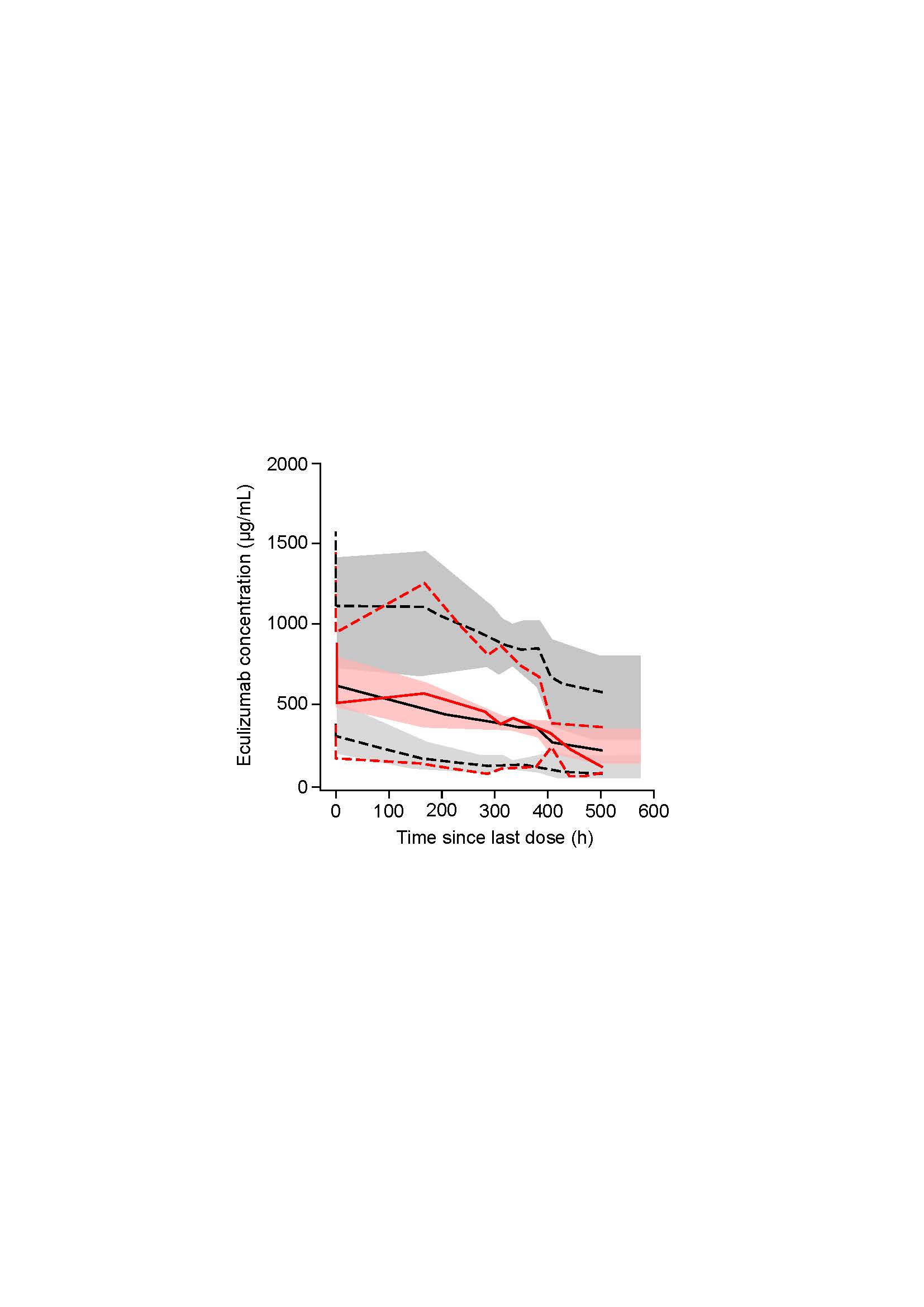


The red solid and dashed lines represent median and 5th–95th percentiles of the observed data. The black solid and dashed lines represent median and 5th–95th percentiles of the simulated data. Shaded areas represent the 90% confidence intervals of the simulated medians (red) and 5th–95th percentiles (gray).

## References

R Core Team. R: A language and environment for statistical computing. Vienna: R Foundation for Statistical Computing (2016). <https://www.r-project.org>

Beal SL, Sheiner LB, Boeckmann AJ. NONMEM Users Guide: Part I–VII. Ellicott City: Icon Development Solutions (1989–2006).

Jonsson EN, Karlsson MO. Automated covariate model building within NONMEM. Pharm Res (1998) 15:1463–8. <https://doi.org/10.1023/a:1011970125687>

# Supplementary Results

**SUPPLEMENTARY TABLE 1** **|** Parameter estimates of the final population-pharmacokinetic model.

|  | **Final model** | | | **Bootstrap** | |
| --- | --- | --- | --- | --- | --- |
| **Parameter** | **Estimate** | **95% CI** | **RSE (%)** | **Median** | **95% CI** |
| CL (L/h) | 0.00749 | 0.00712–0.00788 | 0.5 | 0.00746 | 0.00703–0.0079 |
| V_1_ (L) | 2.37 | 2.13–2.64 | 6.4 | 2.4 | 2.02–2.66 |
| V_2_ (L) | 2.16 | 1.82–2.56 | 11.3 | 2.13 | 1.61–2.62 |
| Q (L/h) | 0.243 | 0.118–0.5 | 26.0 | 0.225 | 0.0371–0.491 |
| Weight effect on CL/Q | 0.908 | 0.780–1.04 | 7.2 | 0.897 | 0.686–1.07 |
| Weight effect on V_1_/V_2_ | 0.594 | 0.421–0.767 | 14.9 | 0.6 | 0.472–0.716 |
| PLEX effect on CL (L/h) | 1.79 | 1.21–2.37 | 16.6 | 1.79 | 1.26–3.91 |
| **Inter-individual variability** | | | | | |
| CL (%CV) | 24.5 | 20.4–28.2 | 15.6 | 24.4 | 20.6–28.0 |
| V_1_ (%CV) | 16.3 | 10.7–20.5 | 28.7 | 15.7 | 11.7–21.1 |
| Proportional error (%CV) | 21.0 | 20.3–21.7 | 1.7 | 20.9 | 19.2–22.9 |

1000 replicates were run for the bootstrap analysis. Shrinkage for CL and V_1_ was 2.8% and 19.9%, respectively.
CI, confidence interval; CL, clearance; CV, coefficient of variation; PLEX, plasmapheresis/plasma exchange; Q, intercompartmental clearance;
RSE, relative standard error; V_1_, volume of distribution in the central compartment; V_2_, volume of distribution in the peripheral compartment.

**SUPPLEMENTARY TABLE 2 |** Eculizumab pharmacokinetic parameters in Asian and non-Asian patients.

|  | **Non-Asian patients (n=58)** | | | | | **Asian patients (n=37)** | | | | |
| --- | --- | --- | --- | --- | --- | --- | --- | --- | --- | --- |
| **Parameter** | **Median** | **5P** | **25P** | **75P** | **95P** | **Median** | **5P** | **25P** | **75P** | **95P** |
| CL (L/h) | 0.0072 | 0.0049 | 0.0062 | 0.0097 | 0.0144 | 0.0058 | 0.0041 | 0.0052 | 0.0068 | 0.0095 |
| V_1_ (L) | 2.42 | 1.78 | 2.15 | 2.84 | 3.39 | 1.93 | 1.58 | 1.72 | 2.19 | 2.87 |
| V_2_ (L) | 2.21 | 1.74 | 1.97 | 2.53 | 2.88 | 1.85 | 1.54 | 1.67 | 2.06 | 2.43 |
| Q (L/h) | 0.252 | 0.175 | 0.212 | 0.31 | 0.377 | 0.193 | 0.145 | 0.164 | 0.226 | 0.292 |
| Terminal half-life^a^ (h) | 424 | 275 | 360 | 496 | 609 | 455 | 324 | 406 | 496 | 594 |
| C_max,ss_ (μg/ml) | 848 | 535 | 678 | 996 | 1,172 | 1,090 | 704 | 863 | 1,220 | 1,418 |
| C_trough,ss_ (μg/ml) | 364 | 152 | 264 | 454 | 593 | 462 | 275 | 409 | 542 | 698 |
| AUC_ss_ (μg·h/ml) | 166,314 | 83,235 | 123,373 | 192,945 | 245,019 | 205,522 | 127,923 | 177,179 | 231,406 | 292,826 |
| C_max,ss_ (μg/ml) – weight normalized | 863 | 650 | 764 | 956 | 1,111 | 838 | 673 | 752 | 901 | 1,043 |
| C_trough,ss_ (μg/ml) – weight normalized | 357 | 192 | 301 | 456 | 546 | 361 | 229 | 306 | 419 | 532 |
| AUC_ss_ (μg·h/ml) – weight normalized | 160,423 | 104,145 | 141,946 | 195,452 | 231,875 | 162,472 | 116,539 | 138,392 | 181,773 | 220,690 |

^a^Terminal half-life was calculated as: (V_1_+V_2_)/CL*ln(2).
5P, 25P, 75P, and 95P, 5th, 25th, 75th, and 95th percentiles; AUC, area under the concentration–time curve within one dosing interval; CL, clearance; C_max_, peak concentration; C_trough_, concentration at the end of the dosage interval; Q, intercompartmental clearance; ss, steady state; V_1_, volume of distribution in the central compartment; V_2_, volume of distribution in the peripheral compartment.

**SUPPLEMENTARY TABLE 3 |** Eculizumab pharmacokinetic parameters in Japanese and non-Japanese patients.

|  | **Non-Japanese patients (n=86)** | | | | | **Japanese patients (n=9)** | | | | |
| --- | --- | --- | --- | --- | --- | --- | --- | --- | --- | --- |
| **Parameter** | **Median** | **5P** | **25P** | **75P** | **95P** | **Median** | **5P** | **25P** | **75P** | **95P** |
| CL (L/h) | 0.0068 | 0.0045 | 0.0057 | 0.0084 | 0.0142 | 0.0055 | 0.0039 | 0.0046 | 0.0061 | 0.008 |
| V_1_ (L) | 2.23 | 1.65 | 1.98 | 2.73 | 3.39 | 1.91 | 1.59 | 1.65 | 2.05 | 2.7 |
| V_2_ (L) | 2.06 | 1.64 | 1.87 | 2.4 | 2.75 | 1.72 | 1.53 | 1.66 | 2.06 | 2.33 |
| Q (L/h) | 0.227 | 0.161 | 0.195 | 0.286 | 0.351 | 0.172 | 0.144 | 0.163 | 0.226 | 0.275 |
| Terminal half-life^a^ (h) | 431 | 268 | 370 | 494 | 604 | 478 | 401 | 419 | 518 | 572 |
| C_max,ss_ (μg/ml) | 882 | 537 | 742 | 1,098 | 1,258 | 1,120 | 776 | 1,070 | 1,230 | 1,478 |
| C_trough,ss_ (μg/ml) | 389 | 156 | 304 | 483 | 631 | 497 | 343 | 430 | 613 | 747 |
| AUC_ss_ (μg·h/ml) | 176,216 | 84,529 | 142,807 | 210,210 | 266,040 | 216,888 | 152,372 | 198,121 | 259,252 | 312,275 |
| C_max,ss_ (μg/ml) – weight normalized | 852 | 652 | 760 | 952 | 1,098 | 847 | 743 | 825 | 881 | 984 |
| C_trough,ss_ (μg/ml) – weight normalized | 357 | 195 | 298 | 443 | 539 | 411 | 314 | 352 | 419 | 477 |
| AUC_ss_ (μg·h/ml) – weight normalized | 160,214 | 106,588 | 137,734 | 188,600 | 228,413 | 173,926 | 142,773 | 153,394 | 181,773 | 200,976 |

^a^Terminal half-life was calculated as: (V_1_+V_2_)/CL*ln(2).
5P, 25P, 75P, and 95P, 5th, 25th, 75th, and 95th percentiles; AUC, area under the concentration–time curve within one dosing interval; CL, clearance; C_max_, peak concentration; C_trough_, concentration at the end of the dosage interval; Q, intercompartmental clearance; ss, steady state; V_1_, volume of distribution in the central compartment; V_2_, volume of distribution in the peripheral compartment.

## Time to First On-Trial Relapse as Determined by the Treating Physician

On-trial relapses as determined by the treating physician occurred in 14/96 of eculizumab-treated patients (15%); the number was equally distributed across exposure quartiles (Supplementary Table 4). Across eculizumab exposure quartiles, there was no separation between relapse-free survival curves for physician-determined relapse (Supplementary Figure 2); however, as for the adjudicated relapse data (see main text), there was again clear separation between the placebo relapse-free survival curve and the curves plotted by eculizumab exposure quartile.

**SUPPLEMENTARY TABLE 4 |** Number of patients with on-trial relapse as determined by the treating physician, by eculizumab exposure quartile.

| **Group** | **Total No. of patients** | **No. of patients with on-trial relapse as determined by the treating physician** |
| --- | --- | --- |
| Patients receiving placebo | 47 | 29 |
| Patients receiving eculizumab |  |  |
| Patients with AUC_ss_ within 1st quartile  (range 58,714–143,644 μg·h/ml) | 24 | 3 |
| Patients with AUC_ss_ within 2nd quartile  (range 144,679–179,692 μg·h/ml) | 24 | 4 |
| Patients with AUC_ss_ within 3rd quartile  (range 181,113–216,888 μg·h/ml) | 23 | 3 |
| Patients with AUC_ss_ within 4th quartile  (range 218,938–331,373 μg·h/ml) | 24 | 4 |

AUC_ss_ was calculated using *post-hoc* pharmacokinetic parameters from the final population-pharmacokinetic model. One eculizumab-treated patient for whom no *post-hoc* pharmacokinetic parameters were obtained was excluded from the analysis.
AUC, area under the concentration–time curve within one dosing interval; ss, steady state.

**SUPPLEMENTARY FIGURE 2 |** Kaplan–Meier survival plots for time to first on-trial relapse as determined by the treating physician, according to eculizumab exposure.


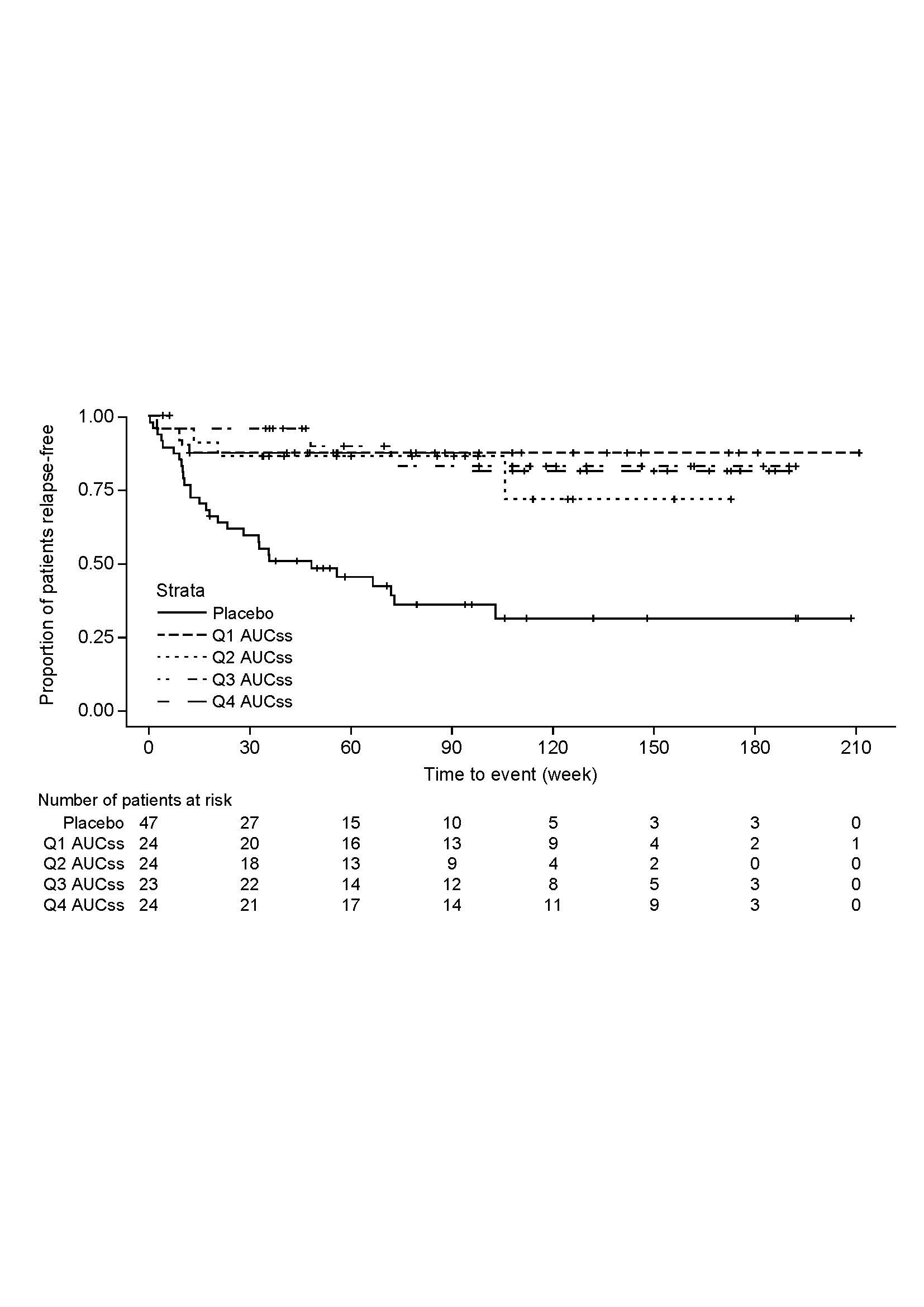


Relapse-free survival curves for each exposure quartile for time to first on-trial relapse in eculizumab-treated patients, as determined by the treating physician. For comparison, the survival curve is also shown for patients receiving placebo. AUC_ss_ is based on the maintenance dose of 1,200 mg eculizumab. One eculizumab-treated patient for whom no *post-hoc* pharmacokinetic parameters were obtained was excluded from the analysis.
AUCss, area under the concentration–time curve within one dosing interval at steady state; Q1, 1st quartile; Q2, 2nd quartile; Q3, 3rd quartile; Q4, 4th quartile.

**SUPPLEMENTARY TABLE 5 |** Incidence of TEAEs occurring in at least 5% of eculizumab-treated patients overall and incidence of TEAESIs, according to eculizumab exposure at steady state

| **Group** | **No. of patients** | **Patients with TEAEs^a^ n [% (95% CI)]** | **Patients with TEAESIs^b^ n [% (95% CI)]** |
| --- | --- | --- | --- |
| Patients receiving placebo | 47 | 40 [85.1 (69.7–94.6)] | 10 [21.3 (9.6–37.7)] |
| Patients receiving eculizumab |  |  |  |
| Total | 95^c^ | 82 (86.3) | 21 (22.1) |
| Patients with AUC_ss_ within 1st quartile  (range 58,714–143,644 μg·h/ml)^d^ | 24 | 21 [87.5 (64.8–98.0)] | 8 [33.3 (13.8–58.1)] |
| Patients with AUC_ss_ within 2nd quartile  (range 144,679–179,692 μg·h/ml)^d^ | 24 | 18 [75.0 (50.4–91.6)] | 6 [25.0 (8.4–49.6)] |
| Patients with AUC_ss_ within 3rd quartile  (range 181,113–216,888 μg·h/ml)^d^ | 23 | 21 [91.3 (69.1–99.3)] | 5 [21.7 (6.3–46.7)] |
| Patients with AUC_ss_ within 4th quartile  (range 218,938–331,373 μg·h/ml)^d^ | 24 | 22 [91.7 (70.2–99.3)] | 2 [8.3 (0.7–29.8)] |

^a^Total number of patients based on TEAEs occurring in at least 5% of patients receiving eculizumab. ^b^Total number of patients, counting all TEAESIs (infections, infusion reactions, serious cutaneous adverse reactions, cardiac disorders, and angioedema) reported. ^c^One eculizumab-treated patient for whom no *post-hoc* pharmacokinetic parameters were obtained was excluded from the analysis. ^d^Based on the maintenance dose of 1,200 mg and calculated as clearance/study-specific maintenance dose.

Abbreviations: AUC_ss_, area under the concentration-time curve within one dosing interval at steady state; TEAE, treatment-emergent adverse event; TEAESI, treatment-emergent adverse event of special interest
